# Roles of retinoic acid-related orphan receptor α in high glucose-induced cardiac fibroblasts proliferation

**DOI:** 10.3389/fphar.2025.1539690

**Published:** 2025-01-30

**Authors:** Wenqing San, Qianyou Zhou, Danning Shen, Danyi Cao, Yun Chen, Guoliang Meng

**Affiliations:** Department of Pharmacology, School of Pharmacy, Nantong University, Nantong, China

**Keywords:** cardiac fibroblasts, retinoic acid-related orphan receptor α, proliferation, necroptosis, oxidative stress

## Abstract

Diabetic cardiomyopathy, characterized by myocardial fibrosis, is a common complication of diabetes. Retinoic acid-related orphan receptor α (RORα) participates in various pathological and physiological cardiovascular processes. The current research aims to elucidate the roles and mechanisms of RORα in high glucose induced cardiac fibroblasts proliferation. Primary neonatal cardiac fibroblasts were isolated from Sprague-Dawley rats, and pre-administrated with RORα antagonist SR3335 (20 µM) or RORα agonist SR1078 (10 µM) followed by the stimulation with normal glucose (5.5 mM) or high glucose (33.3 mM) respectively. Lactate Dehydrogenase (LDH) release into culture medium, cellular adenosine-triphosphate (ATP), and cell number were detected. Expressions of Collagen I, Collagen III, proliferating cell nuclear antigen (PCNA), α-smooth muscle actin (α-SMA), receptor-interacting protein kinase 1 (RIPK1) and receptor-interacting protein kinase 3 (RIPK3) were evaluated. The extent of oxidative stress was also assessed. Our study found that high glucose elevated LDH release, reduced cellular ATP production, increased cells numbers, elevated expression of Collagen I, Collagen III, PCNA, α-SMA, RIPK1 and RIPK3, decreased mitochondrial membrane potential, strengthened intensity of dihydroethidium (DHE) and MitoSOX fluorescence. Above effects were all further exacerbated by SR3335 but significantly reversed by SR1078. In conclusion, RORα antagonist SR3335 promoted cell injury and proliferation, enhanced collagen synthesis, facilitated oxidative stress and necroptosis in cardiac fibroblasts with high glucose stimulation, whereas RORα agonist SR1078 showed opposing effects. Our study proposed RORα as a novel target against high glucose-induced cardiac fibroblasts proliferation, which is beneficial to clarify ideal therapeutic implication for diabetic cardiomyopathy.

## 1 Introduction

Diabetic cardiomyopathy (DC) was originally characterized as the presence of structural or functional abnormalities of the myocardium associated with diabetes mellitus (DM) in the absence of hypertension, coronary heart disease, and/or obesity (Song et al., 2021). However, this characterization lacks robust evidence as only a limited number of diabetic patients meet these criteria, rendering its clinically impractical. Recently, the Heart Failure Association of the European Society of Cardiology (ESC), in collaboration with the Working Group on Myocardial and Pericardial Diseases, has published a consensus statement proposing that DC should be defined as the presence of myocardial diastolic and/or systolic dysfunction related to diabetes (Seferović et al., 2024). From the standpoint of heart failure progression, the asymptomatic functional and structural cardiac anomalies in patients with DC can be considered as precursors to heart failure. Nonetheless, therapeutic options for DC remain limited in clinical practice. Furthermore, the role of glycemic control in the prevention of heart failure among diabetic patients is not well understood. Some studies have indicated a U-shaped relation between blood glucose levels and the incidence of heart failure, suggesting that glycemic control alone may be insufficient to prevent the progression of DC to heart failure (Parry et al., 2015). Consequently, clarifying pathogenesis of DC and exploring rational and effective treatment strategies will be beneficial in prevention and management of DC.

Myocardial fibrosis is a prominent pathological feature observed in DC, manifested primarily as an excessive accumulation of collagen fibers, a marked increase in collagen content or abnormal alterations in collagen composition. These changes lead to an elevated number of cardiac fibroblasts within the extracellular matrix (ECM) of the myocardium (Frangogiannis, 2022). Cardiac fibroblasts play a crucial role in maintaining ECM homeostasis. Upon activation by several stimuli or damaging factors, such as ischemia, pressure overload, metabolic disorders, and neurohormonal release, cardiac fibroblasts differentiate into myofibroblasts, which are instrumental in driving pathological cardiac remodeling (Liu T. et al., 2024; González et al., 2024; Yu et al., 2024; Zhang et al., 2023a). Myofibroblasts exhibit proliferative capabilities and contribute to ECM turnover and collagen deposition. However, there remains a significant gap in effective strategies to prevent excessive proliferation of cardiac fibroblasts in the context of DC.

Necroptosis, a form of programmed cell death identified as an alternative to apoptosis following the binding death structural domains to receptor, playing a significant role in myocardial hypertrophy, myocardial infarction, atherosclerosis, and neurodegenerative diseases (Cai et al., 2024; Zhang et al., 2023b; Sheng et al., 2023; Cao and Mu, 2021; Khan et al., 2021). Necroptosis is characterized by rupture of cell membranes, swelling of organelles, enlargement of cell volume, and breakdown of cytoplasm and nucleus, while exhibiting minimal alterations in nuclear chromatin. Increasingly, studies have shown that the necroptotic pathway is mediated by the canonical death receptor comprising of receptor-interacting protein kinase 1 (RIPK1) and receptor-interacting protein kinase 3 (RIPK3) (Zhou et al., 2019; Shao et al., 2021). Active RIPK1 participates in the formation of oligomeric complexes that involve caspase-8, caspase-10 and Fas-associated protein with death domain (FADD). In detail, RIPK1 phosphorylates RIPK3, which subsequently phosphorylates mixed lineage kinase domain-like protein (MLKL), leading to the formation of necrosomes. Following this, MLKL oligomers translocate to phosphatidylinositol phosphate (PIP)-rich region of plasma membrane, resulting in the formation of large pores, causing a substantial influx of ions, lysis of the cell membrane, permeabilization of lysosomal membrane and uncontrolled release of intracellular contents, culminating in necroptosis (Koerner et al., 2024; Liu S. et al., 2024). Moreover, our previous studies have established a correlation between necroptosis and mitochondrial dysfunction, oxidative stress, and inflammation during DC (Song et al., 2021; Gong et al., 2022; Zhang S. et al., 2023), indicating that necroptosis inhibition may protect against cardiac fibroblasts proliferation in DC.

Retinoic acid-related orphan receptor (ROR) is classified within nuclear hormone receptor superfamily, which integrates nutritional, pathophysiological, hormonal signaling and gene regulation (Zheng et al., 2024; Sajinovic and Baier, 2023). Three primary isoforms in mammals are recognized: RORα, RORβ, and RORγ, each of which is capable of forming multiple variants through selective splicing. RORα has been associated with various functions, including neurodevelopment, cellular differentiation, immunoregulation, metabolism, and the regulation of circadian rhythms. Recent studies have revealed that RORα exerts a protective impact against cardiovascular disorders such as myocardial hypertrophy, myocardial ischemia-reperfusion injury, and atherosclerosis (Chen et al., 2023). Our previous research demonstrated a significant reduction in RORα expression in diabetic hearts, and lack of RORα exacerbated diabetes-induced systolic dysfunction and cardiac remodeling (Zhang S. et al., 2023). These findings suggest that RORα may possess an inhibitory role in DC. However, the specific influence of RORα on cardiac fibroblast proliferation during DC remains inadequately understood.

Therefore, in our current study, the primary cardiac fibroblasts were isolated and subsequently subjected to high glucose stimulation. The study aimed to elucidate the effects and potential mechanisms of RORα antagonist and RORα agonist on cardiac proliferation, with a focus on oxidative stress and necroptosis. It is conducive to provide innovative insights for clinical prevention and treatment of DC.

## 2 Materials and methods

### 2.1 Culture and treatment of primary cardiac fibroblasts

Hearts from Sprague-Dawley rats aging one to 3 days were excised and rapidly taken off using sterilized surgical scissors. After rinsing three times in cold phosphate buffered saline (PBS) solution, the hearts were cut into approximately 1–3 mm^3^ cubes and transferred into a 50 mL of conical bottle. About 1.5–2.0 mL of Dulbecco’s modified eagle medium (DMEM, Wisent Inc., Montreal, QC, Canada) having trypsin was added into the conical bottle placed on an incubator with shaking for 5 min at 37°C to start digestion. The first digestion’s supernatant was discarded. Then, the precipitate underwent further digestion for 3 min at a time and repeated for about 10 times. All digested supernatants were collected into a beaker containing DMEM with 10% fetal bovine serum (FBS, Gibco, Thornton, NSW, Australia). The cell suspension after filtering with a cell sieve was centrifuged in a centrifuge tube for 5 min at 1,200 r/min. Following removing the supernatant, the cells in the precipitate were re-suspended and inoculated into a new culture dish with DMEM having 10% FBS. The cells were placed at 37°C in 5% CO_2_ incubator, and the differential adhesion method to acquire cardiac fibroblasts was performed. In detail, culture medium containing cardiomyocytes was removed after cells had adhered to the plate for 180 min. The cardiac fibroblasts that remained attached to the plate were digested and cultured in fresh DMEM having 10% FBS. The cardiac fibroblasts were sub-cultured basing on their growth conditions, and the cardiac fibroblasts of 3rd to 4th generation were seeded into plates in the present study. After starvation for 12 h, the cardiac fibroblasts were pre-administrated with RORα antagonist SR3335 (5, 10, 20 and 40 μM, MedChemexpress, Rahway, NJ, United States) or RORα agonist SR1078 (2.5, 5, 10 and 20 μM, MedChemexpress) followed by 48 h of stimulation with normal glucose (NG) or high glucose (HG) respectively (Liang et al., 2021; Chen D. et al., 2024; Wahyuni et al., 2021; Zhang Y. et al., 2022; Xiong et al., 2020; Shen et al., 2023). Cardiac fibroblasts under the normal glucose (5.5 mM) group and high glucose (11.1 mM, 22.2 mM and 33.3 mM) group were exposed to 27.8 mM, 22.2 mM, 11.1 mM mannitol and 0 mM mannitol respectively to balance the osmotic pressure (Gong et al., 2022; Zhang S. et al., 2023; Tian et al., 2021; Lu et al., 2023).

The study was conducted according to National Institutes of Health guidelines for the Care and Use of Laboratory Animals, and approved by Committee of Nantong University (approval no. S20210227-011 on 27 February 2021). The study was conducted in accordance with the local legislation and institutional requirements.

### 2.2 Lactate dehydrogenase (LDH) release detection

After treatment, the centrifugation was made for cell culture medium at 400 g for 5 min. The supernatants of 120 μL were collected and transferred to 96-well plate followed by incubation at 25°C for 30 min with 60 μL of LDH test solution (Beyotime, Shanghai, China) without light. The absorbance at 490 nm, representing LDH release level, were recorded by microplate-reader (BioTek, Winooski, VT, United States) and standardized by the value obtained from the normal glucose group value.

### 2.3 Adenosine-triphosphate (ATP) level measurements

After treatment, 100 μL ATP assay reagent (Beyotime, Shanghai, China) was utilized to incubate cardiac fibroblasts at 25°C for 10 min. Then, microplate-reader was employed to record the luminescence intensity. The relative ATP levels were standardized by the value obtained from the normal glucose group.

### 2.4 Cell counting kit-8 (CCK-8) assay

After treatment, 10 μL CCK-8 reagent (Beyotime, Shanghai, China) was added to cardiac fibroblasts in 96-well plates and incubated at 37°C for 1 h without light. The optical density (OD), which correlates with the cell number, was recorded for each sample by a microplate-reader at 450 nm.

### 2.5 EdU (5-ethynyl-2′-deoxyuridine) staining

After treatment, EdU (50 μM, RiboBio, Guangzhou, China) was added to cardiac fibroblasts in 24-well plates and incubated for 2 h without light. Next, the cells were washed twice with PBS. Then, PBS containing 4% paraformaldehyde was used to fix the cells for 30 min. Glycine (2 mg/mL) was then added and the mixture was agitated on a shaker for 5 min. After washing, EdU penetrant (PBS containing 0.5% TrixonX-100) was used to incubate the cells for 10 min, and washed by PBS once for 5 min. Cells were incubated with EdU penetrant once again for an additional 10 min after the application of Apollo fluorescence staining solution for 30 min 4′,6-diamidino-2-phenylindole (DAPI, blue) was used to stain the nuclei. EdU red fluorescence was monitored and imaged with a confocal laser microscope (Leica, Wetzlar, Germany). ImageJ software was employed to count the EdU positive cells.

### 2.6 Immunofluorescence staining

After treatment, the cardiac fibroblasts in 24-well plates were fixed at 25°C for 30 min with immunofluorescence fixative. Following fixation, the cells were washed with PBS and incubated for 1 h with blocking solution. The primary antibodies, including RORα (1:200, Abcam, Cambridge, United Kingdom), α-smooth muscle actin (α-SMA, 1:1,000, Boster Biological Technology, Dublin, CA, United States), Collagen I and Collagen III (1:200, Proteintech, Rosemont, IL, United States) were applied and incubated overnight at 4°C. PBS was used for washing followed by the incubation of cells with Alexa Fluor 488 (green) or Cy3 (red) conjugated IgG dilution (1:500, Beyotime, Shanghai, China) on a shaker for 2 h without light at 25°C. DAPI (blue) was used to stain the nuclei. The fluorescence was monitored and imaged with a confocal laser microscope.

### 2.7 Dihydroethidium (DHE) staining

After treatment, DHE (2 μM, Beyotime, Shanghai, China) was added to incubate the cardiac fibroblasts at 37°C without light for 30 min in 24-well plates placed in oven. DAPI was employed to stain the nuclei. Red fluorescence reflecting superoxide anion levels were monitored and imaged with a confocal laser microscope.

### 2.8 MitoSOX staining

After treatment, MitoSOX Red (5 μM, Yeasen, Shanghai, China) and MitoTracker Green (200 nM, Beyotime, Shanghai, China) were added to incubate cardiac fibroblasts at 37°C in 24-well plates for 20 min without light in an oven. DAPI was employed to stain the nuclei. Red fluorescence reflecting mitochondria reactive oxygen species (ROS) levels were monitored and imaged with a confocal laser microscope.

### 2.9 JC-1 staining

After treatment, JC-1 (5,5′,6,6′-Tetrachloro-1,1′,3,3′-tetraethyl-imidacarbocyanine iodide) working solution (Beyotime, Shanghai, China) was added to incubate cardiac fibroblasts in 24-well plates at 37°C for 20 min in an oven without light. DAPI was employed to stain the nuclei. JC-1 monomers show green fluorescence reflecting impaired mitochondria and JC-1 aggregates show red fluorescence reflecting normal mitochondria with less and higher mitochondrial membrane potentials, respectively. They were detected and imaged with a confocal laser microscope.

### 2.10 Quantitative real-time PCR

TRIzol reagent (Takara, Kyoto, Japan) was used to extract RNA from cardiac fibroblast and reverse transcription was performed. Then, SYBR Green qPCR mixture (Takara) was employed to amplify cDNA in the Real-time PCR systems (ABI 7500, Carlsbad, CA, United States). The sequences of primers (Sangon Biotech, Shanghai, China) were as follows: rat α-SMA mRNA (F, 5′-CAT​CAG​GAA​CCT​CGA​GAA​GC-3′ and R, 5′-TCG​GAT​ACT​TCA​GGG​TCA​GG-3′), rat Collagen I mRNA (F, 5′-AGG​GTC​ATC​GTG​GCT​TCT​CT-3′ and R, 5′-CAG​GCT​CTT​GAG​GGT​AGT​GT-3′), rat Collagen III mRNA (F, 5′-AGC​GGA​GAA​TAC​TGG​GTT​GA -3′ and R, 5′-GAT​GTA​ATG​TTC​TGG​GAG​GC-3) and 18S mRNA (F, 5′-AGT​CCC​TGC​CCT​TTG​TAC​ACA-3′ and R, 5′-CGA​TCC​GAG​GGC​CTC​ACT​A-3′). Standardization was made for the experimental Ct values by those in normal glucose group.

### 2.11 Western blot

After washing with PBS 2–3 times, lysis solution was added into the cardiac fibroblasts and incubated for 40 min on ice. Then, cells were scraped off using a cell spatula, collected into centrifuge tubes and continued to lysis for an additional 40 min. Next, the cells were centrifuged with 12,000 rpm at 4°C for 15 min to collect the supernatant and stored at −80°C for subsequent experiments. Protein quantification (BCA method) was made to determine the protein concentration and sample volume for measurement was calculated.

Next, sodium dodecyl sulfate (SDS)-polyacrylamide gel electrophoresis (PAGE) was used to separate the proteins followed by transferring to polyvinylidene fluoride (PVDF) membrane. Then, 5% non-fat milk was employed to incubate the membranes for 2 h at 25°C. After washing for 10 min by Tris-buffered saline Tween-20 (TBST), RORα (1:1,000, Abcam, Cambridge, United Kingdom), proliferating cell nuclear antigen (PCNA, 1:1,000, ABclonal, Wuhan, China), α-SMA (1:2000, Boster Biological Technology, Dublin, CA, United States), Collagen I and Collagen III (1:200, Proteintech, Rosemont, IL, United States), RIPK1 and RIPK3 (1:1,000, Cell Signaling Technology, Danvers, MA, United States), GAPDH (1:5,000, Sigma-Aldrich, St. Louis, MO, United States), and β-tubulin (1:3,000, CMCTAG, Milwaukee, WI, United States) antibodies were incubated at 4°C overnight. Next day, TBST was used to wash the membrane three times for 10 min each. A secondary antibody was then added followed by incubation for 2 h at 25°C on a shaker. Finally, blots were visualized using an enhanced chemiluminescence (ECL, Thermo Fisher Scientific Inc., Rockford, IL, United States) solution.

### 2.12 Statistical analysis

The data were presented as mean ± standard deviation (SD), and statistically evaluated by one-way ANOVA followed by the Student-Newman-Keuls test with Stata 15.0. *p-*value of <0.05 was set as statistically significant.

## 3 Results

### 3.1 High glucose promoted cell proliferation but inhibted RORα expressions in cardiac fibroblasts

**FIGURE 1:**
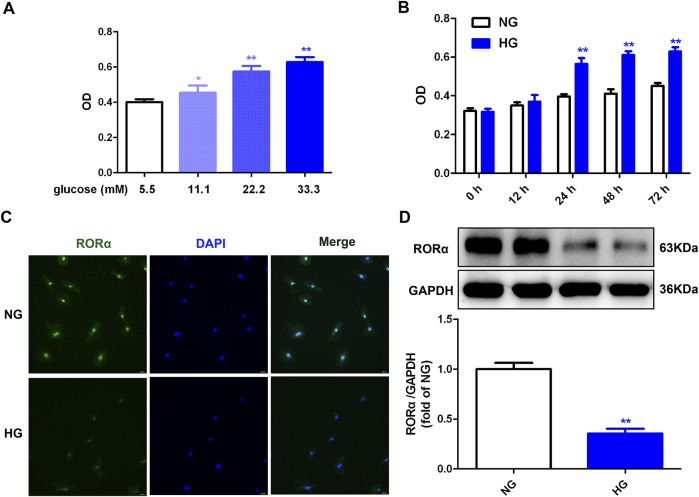
High glucose promoted cell proliferation but inhibted RORα expressions in cardiac fibroblasts. **(A)** After stimulation with glucose of different concentration (5.5, 11.1, 22.2 and 33.3 mM) and mannitol with different concentration (27.8 mM, 22.2 mM, 11.1 mM and 0 mM respectively) for 48 h, OD value obtained from CCK-8 assay was measured. ^*^ *p <* 0.05, ^**^ *p <* 0.01 verses 5.5 mM glucose, n = 6. **(B)** After stimulation with normal glucose (NG, glucose 5.5 mM and mannitol 27.8 mM) or high glucose (HG, 33.3 mM) for different times (0 h, 12 h, 24 h, 48 h and 72 h), OD value obtained from CCK-8 assay was measured. ^**^ *p <* 0.01 verses 5.5 mM glucose with the same stimulation time, n = 6. **(C)** After stimulation with NG or HG for 48 h, RORα was immunofluorescence stained with Alexa Fluor 488 (green) conjugated IgG. The nuclei were stained with DAPI (blue). Bar = 50 μm. **(D)** The protein expression of RORα was measured by Western blot. GAPDH was serviced as a control. ^**^ *p <* 0.01 verses NG, n = 6.

Initially, a concentration-response curve was established to assess the relation between glucose concentration and cell number. The data demonstrated that glucose concentrations of 11.1, 22.2 and 33.3 mM significantly increased cell number, with the most pronounced effects observed at a concentration of 33.3 mM (Figure 1A). Then, a time-dependent experiment showed that stimulation with 33.3 mM glucose for durations of 24 h, 48 h and 72 h increased cell numbers, with the maximum enhancement begining at 48 h (Figure 1B). Therefore, a 48 h exposure to 33.3 mM glucose was selected for subsequent experiments aimed at promoting cell proliferation, consistent with previous studies (Gong et al., 2022; Zhang S. et al., 2023; Tian et al., 2021; Lu et al., 2023).

Our previous research confirmed that high glucose decreased RORα expression in cardiomyocytes (Zhang S. et al., 2023). In alignment with these findings, the current study confirmed that high glucose also reduced RORα expression in cardiac fibroblasts (Figures 1C, D). To elucidate the role of RORα in cardiac fibroblast proliferation, the effects of RORα antagonist and RORα agonist on primary cardiac fibroblasts with high glucose stimulation were further investigated.

### 3.2 SR3335 promotes cell injury and proliferation in high glucose stimulated cardiac fibroblasts

**FIGURE 2:**
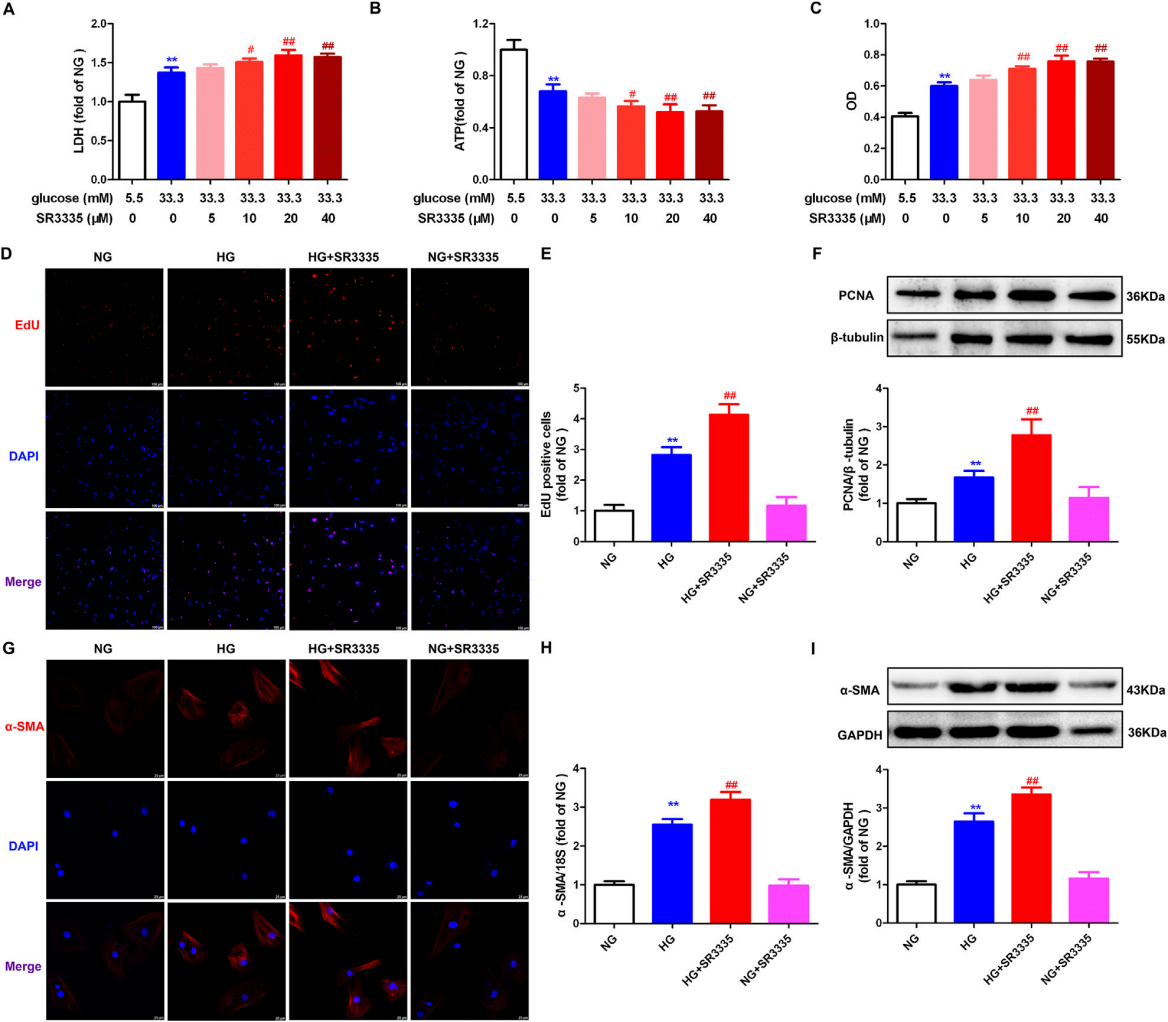
SR3335 promoted cell injury and proliferation in high glucose stimulated cardiac fibroblasts. **(A–C)** After pre-administration with different concentration of SR3335 (5 μM, 10 μM, 20 μM and 40 µM) for 4 h, the cardiac fibroblasts were stimulated with normal glucose (NG, glucose 5.5 mM and mannitol 27.8 mM) or high glucose (HG, 33.3 mM) for 48 h. LDH release in the medium **(A)**, cellular ATP in the cardiac fibroblasts **(B)**, and OD value obtained from the CCK-8 assay **(C)** were measured. ^**^ *p* < 0.01 verses 5.5 mM glucose; ^#^ *p* < 0.05, ^##^ *p* < 0.01 verses 33.3 mM glucose, n = 6. **(D, E)** After pre-administration with SR3335 (20 µM) for 4 h, the cardiac fibroblasts were stimulated with NG or HG for 48 h. EdU staining with red fluorescent was performed to evaluate cardiac fibroblasts proliferation. Bar = 100 μm. EdU positive cells were quantitatively analyzed. **(F)** The protein expression of PCNA was measured by Western blot. β-tubulin was serviced as a control. **(G)** α-SMA was immunofluorescence stained with Cy3 (red) conjugated IgG. The nuclei were stained with DAPI (blue). Bar = 25 μm. **(H)** The mRNA expression of α-SMA was measured by Real-time PCR. **(I)** The protein expression of α-SMA was measured by Western blot. GAPDH was serviced as a control. ^**^ *p* < 0.01 verses NG; ^##^ *p* < 0.01 verses HG, n = 6.

To evaluate the impact of RORα on cell injury induced by high glucose, LDH release and ATP level were measured. And cardiac fibroblast number was assessed through OD obtained from the cell CCK-8 assay. The data demonstrated that compared to high glucose stimulation alone, RORα antagonist SR3335 at different concentration (10 μM, 20 μM and 40 µM) further increased LDH release in the medium, reduced the cellular ATP production but enhanced OD value in cardiac fibroblasts (Figures 2A–C). These findings suggested that SR3335 promoted cell injury and increased cell number in high glucose stimulated cardiac fibroblasts. Notably, the most pronounced effects were observed at a concentration of 20 μM, which was selected for subsequent experiments.

EdU is capable of infiltrating the DNA that is newly synthesized. Thus, EdU staining is a sensitive and effective method for evaluating cell proliferation (Zhang et al., 2021; Zhang Y. et al., 2024). It was found that EdU positive cells were enhanced in response to high glucose stimulation, with further enhancement by SR3335 (Figures 2D, E). PCNA, a crucial protein associated with DNA polymerase and cell proliferation (He et al., 2024). Western blot showed increased PCNA expression after stimulation by high glucose was further augmented by SR3335 (Figure 2F). Additionally, Western blot, Real-time PCR, and immunofluorescence demonstrated that α-SMA, another sensitive indicator of cell proliferation, was enhanced after stimulation by high glucose, with further promotion by SR3335 (Figures 2G–I). Taken together, SR3335 promoted cell proliferation in high glucose stimulated cardiac fibroblasts.

### 3.3 SR3335 enhances synthesis of collagen in high glucose stimulated cardiac fibroblasts

**FIGURE 3:**
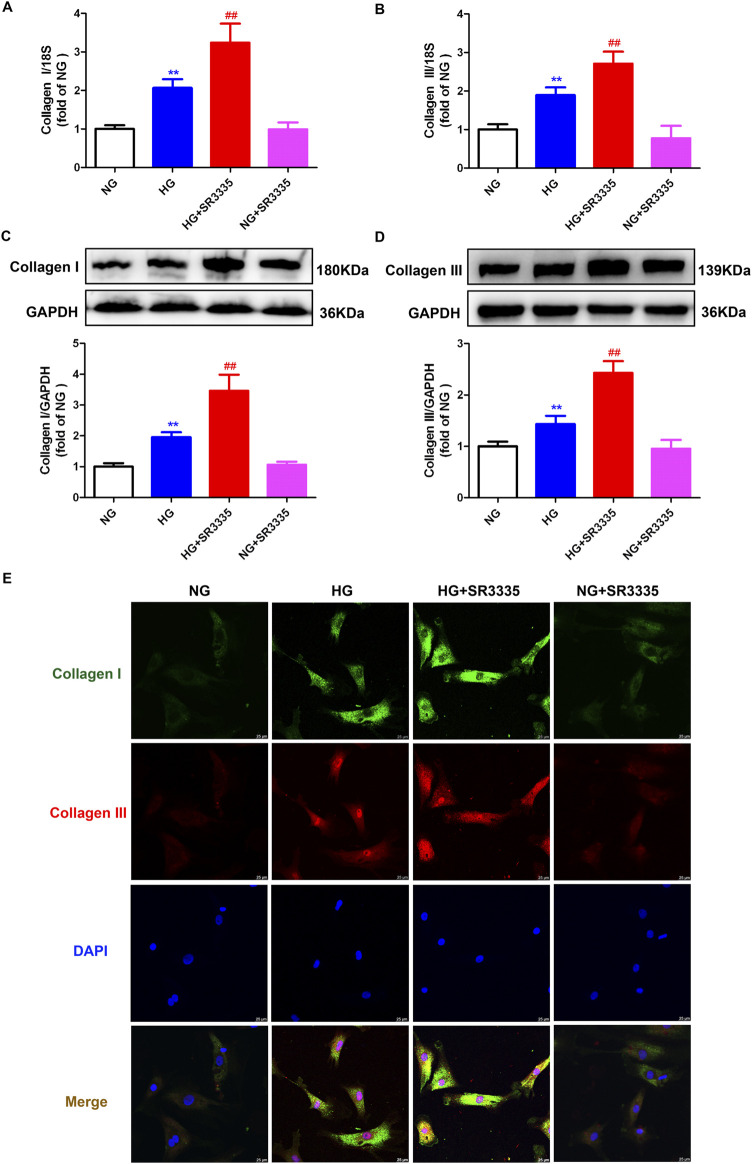
SR3335 enhanced collagen synthesis in high glucose stimulated cardiac fibroblasts. After pre-administration with SR3335 (20 µM) for 4 h, the cardiac fibroblasts were stimulated with normal glucose (NG, glucose 5.5 mM and mannitol 27.8 mM) or high glucose (HG, 33.3 mM) for 48 h **(A, B)** The mRNA expressions of Collagen I and Collagen III were measured by Real-time PCR. **(C, D)** The protein expressions of Collagen I and Collagen III were measured by Western blot. GAPDH was serviced as a control. **(E)** Collagen I and collagen III were immunofluorescence stained with Alexa Fluor 488 (green) and Cy3 (red) conjugated IgG, respectively. The nuclei were stained with DAPI (blue). Bar = 25 μm ^**^ *p* < 0.01 verses NG; ^##^ *p* < 0.01 verses HG, n = 6.

Obviously, increased cardiac fibroblasts during cell proliferation will secret a large amount of collagen. Real-time PCR, Western blot, and immunofluorescence demonstrated that Collagen I and Collagen III, two predominant types of fibroblasts in the myocardium, were enhanced after stimulation by high glucose. This effect was further augmented by SR3335 (Figure 3), suggesting that SR3335 enhanced synthesis of collagen in high glucose stimulated cardiac fibroblasts.

### 3.4 SR3335 facilitates oxidative sstress in high glucose stimulated cardiac fibroblasts

**FIGURE 4:**
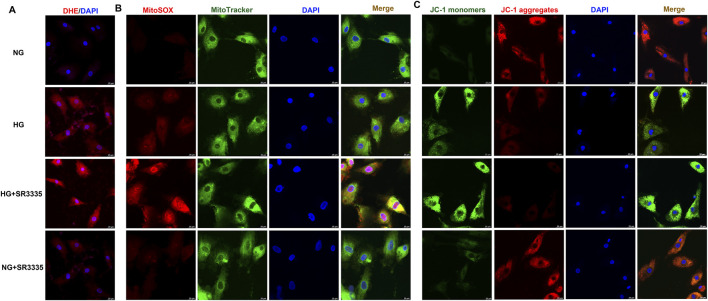
SR3335 facilitated oxidative stress in high glucose stimulated cardiac fibroblasts. After pre-administration with SR3335 (20 µM) for 4 h, the cardiac fibroblasts were stimulated with normal glucose (NG, glucose 5.5 mM and mannitol 27.8 mM) or high glucose (HG, 33.3 mM) for 48 h **(A)** DHE staining with red fluorescent was performed to measure superoxide anion production in cardiac fibroblasts. Bar = 25 μm. **(B)** MitoSOX staining with red fluorescent was performed to measure Mitochondrial ROS production. MitoTracker with green fluorescent was stained to co-localize Mitochondria. Bar = 25 μm. **(C)** Mitochondrial membrane potential was measured by JC-1 staining. Bar = 25 μm.

Reported studies suggested that oxidative stress played a vital role in cardiac fibroblasts proliferation (Zhang Q. et al., 2022; Li et al., 2023). The present research found that red fluorescence of DHE was significantly enhanced after high glucose stimulation, which was further amplified by SR3335 (Figure 4A). This suggested that SR3335 boosted cellular superoxide anion in high glucose stimulated cardiac fibroblasts. ROS in the mitochondria was further measured using MitoSOX staining. Similarly, MitoSOX fluorescence was dramatically strengthened by high glucose, with further enhancement by SR3335 (Figure 4B).

The impairment of mitochondrial membrane potential not only leads to cell injury but also induces oxidative stress (Drăgoi et al., 2024; Chen S. et al., 2024). JC-1 staining demonstrated that JC-1 monomers’ green fluorescence was enhanced while JC-1 aggregates’ red fluorescence was attenuated in cardiac fibroblasts with high glucose stimulation, indicating that high glucose had impaired the mitochondrial membrane potential. SR3335 further enhanced green fluorescence but alleviated red fluorescence of JC-1 staining for high glucose stimulated cardiac fibroblasts (Figure 4C). Collectively, SR3335 facilitated oxidative stress in high glucose stimulated cardiac fibroblasts.

### 3.5 SR3335 promotes necroptosis in high glucose stimulated cardiac fibroblasts

**FIGURE 5:**
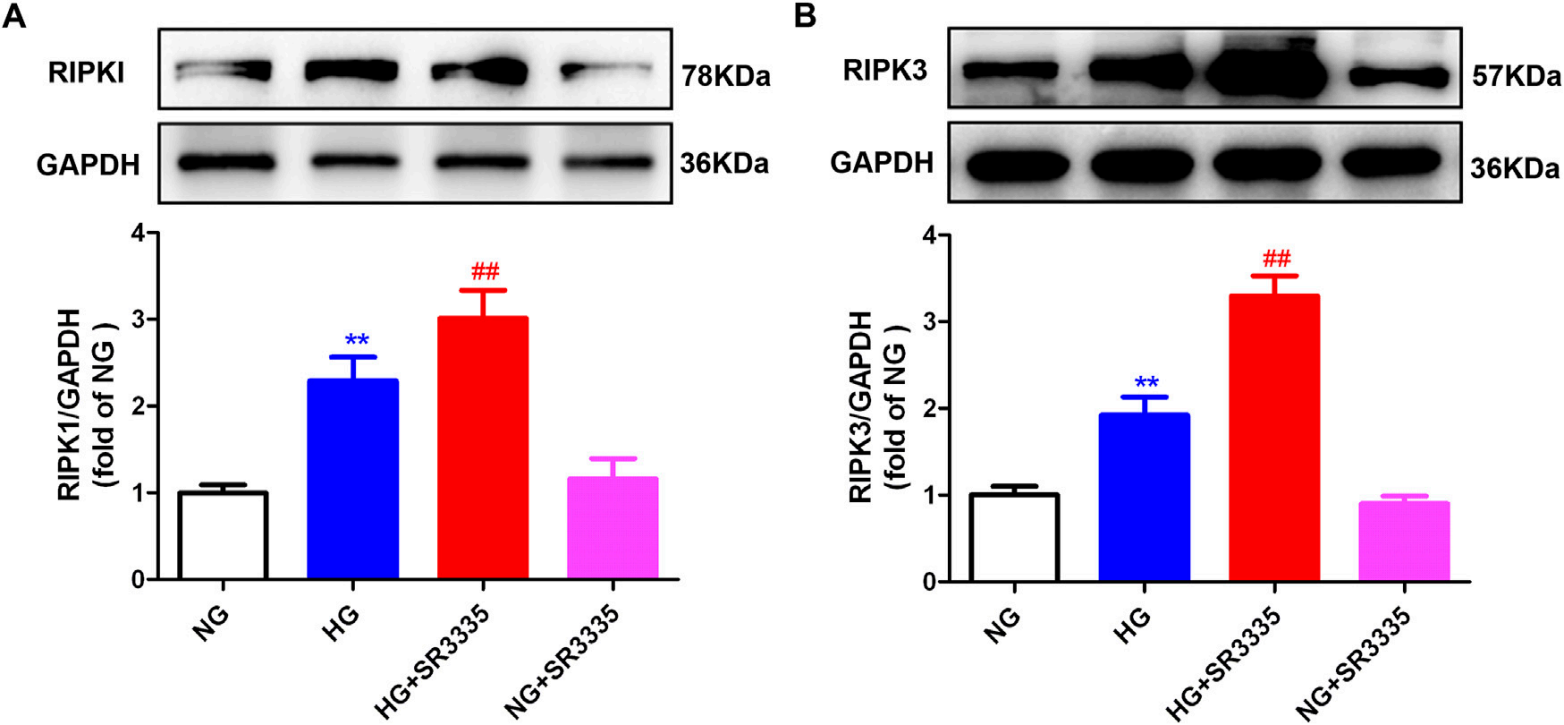
SR3335 promoted necroptosis in high glucose stimulated cardiac fibroblasts. After pre-administration with SR3335 (20 µM) for 4 h, the cardiac fibroblasts were stimulated with normal glucose (NG, glucose 5.5 mM and mannitol 27.8 mM) or high glucose (HG, 33.3 mM) for 48 h. Expression of RIPK1 **(A)** and RIPK3 **(B)** protein was measured by Western blot. GAPDH was serviced as a control. ^**^ *p* < 0.01 verses NG; ^##^ *p* < 0.01 verses HG, n = 6.

A significant release of cellular content following cell injury can trigger necroptosis, thereby aggravating cell damage and promoting cell proliferation (Zhou et al., 2024). This current work demonstrated that RIPK1 and RIPK3 expressions, two hallmark proteins associated with necroptosis, were enhanced after stimulation by high glucose. This effect was further augmented by SR3335 (Figure 5), suggesting that SR3335 promoted necroptosis in high glucose stimulated cardiac fibroblasts.

### 3.6 SR1078 attenuates cell injury and proliferation in high glucose stimulated cardiac fibroblasts

**FIGURE 6:**
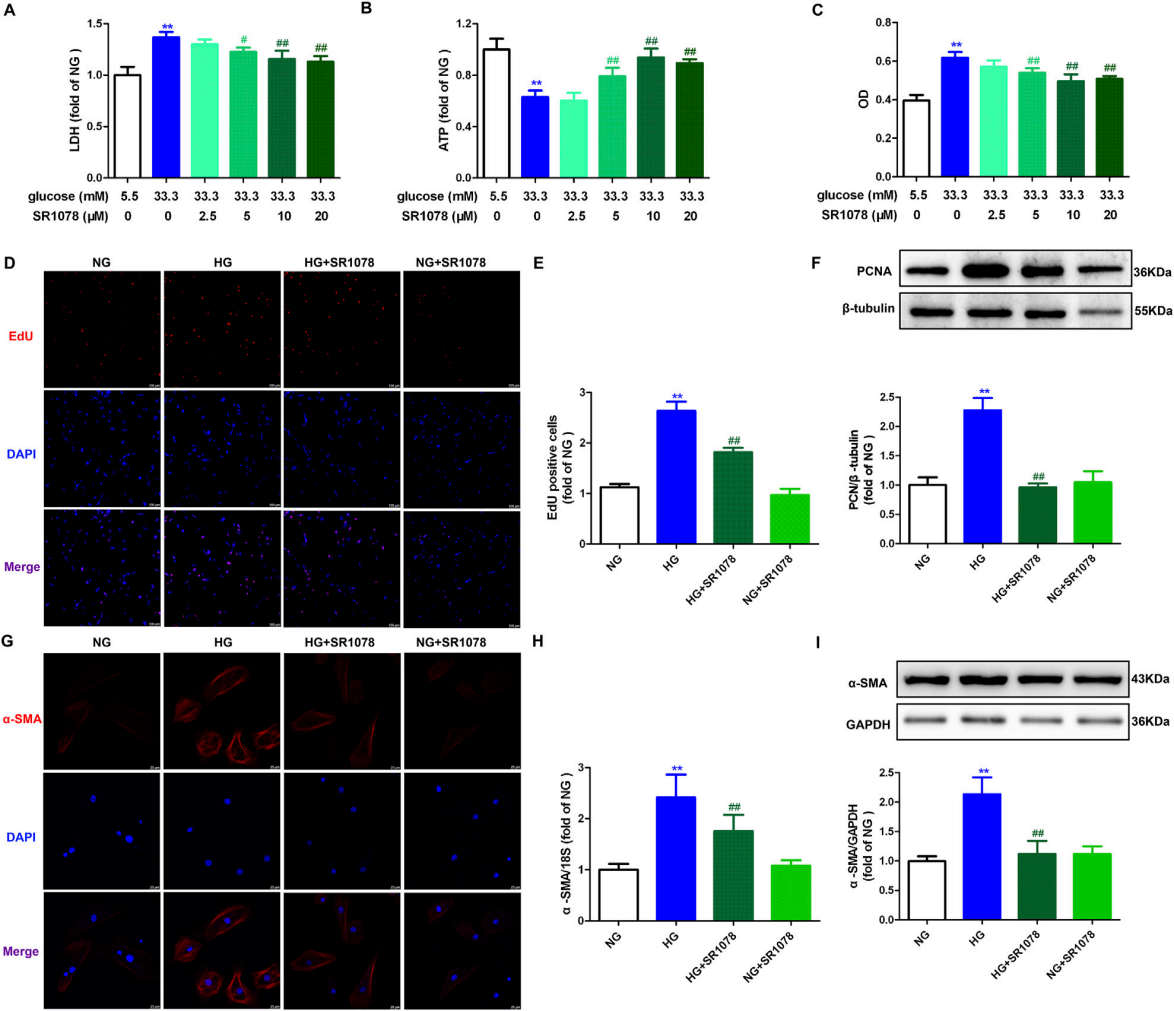
SR1078 attenuated cell injury and proliferation in high glucose stimulated cardiac fibroblasts. **(A–C)** After pre-administration with different concentration of SR1078 (2.5 µM, 5 μM, 10 μM and 20 µM) for 4 h, the cardiac fibroblasts were stimulated with normal glucose (NG, glucose 5.5 mM and mannitol 27.8 mM) or high glucose (HG, 33.3 mM) for 48 h. LDH release in the medium **(A)**, cellular ATP in the cardiac fibroblasts **(B)**, and OD value obtained from CCK-8 assay **(C)** were measured. ^**^ *p* < 0.01 verses 5.5 mM glucose; ^#^ *p* < 0.05, ^##^ *p* < 0.01 verses 33.3 mM glucose, n = 6. **(D, E)** After pre-administration with SR1078 (10 µM) for 4 h, the cardiac fibroblasts were stimulated with NG or HG for 48 h. EdU staining with red fluorescent was performed to evaluate cardiac fibroblasts proliferation. Bar = 100 μm. EdU positive cells were quantitatively analyzed. **(F)** The protein expression of PCNA was measured by Western blot. β-tubulin was serviced as a control. **(G)** α-SMA was immunofluorescence stained with Cy3 (red) conjugated IgG. The nuclei were stained with DAPI (blue). Bar = 25 μm. **(H)** The mRNA expression of α-SMA was measured by Real-time PCR. **(I)** The protein expression of α-SMA was measured by Western blot. GAPDH was serviced as a control. ^**^ *p* < 0.01 verses NG; ^##^ *p* < 0.01 verses HG, n = 6.

The aforementioned data verified that RORα antagonist SR3335 promoted oxidative stress and necroptosis to accelerate proliferation after high glucose stimulation in cardiac fibroblasts. Nonetheless, the potential of RORα agonists to resist proliferation in cardiac fibroblasts under similar conditions remains to be elucidated. Our study demonstrated that RORα agonist SR1078 at different concentration (5 μM, 10 μM and 20 µM) significantly reduced LDH release in the medium, elevated the cellular ATP production and decreased OD value in cardiac fibroblasts with high glucose stimulation (Figures 6A–C). These findings suggested that SR1018 attenuated cell injury and decreased cell number in high glucose stimulated cardiac fibroblasts. Notably, SR1078 exhibited the most reversal effects at a concentration of 10 μM, which was chosen for subsequent experiments.

Additionally, the enhanced number of EdU positive cells were restrained by SR1078 in high glucose stimulated cardiac fibroblasts (Figures 6D, E). Moreover, elevated expressions PCNA and α-SMA were also suppressed by SR1078 in these cells (Figures 6F–I). Taken together, SR1078 attenuated cell proliferation in high glucose stimulated cardiac fibroblasts.

### 3.7 SR1078 reduces synthesis of collagen inHigh glucose stimulated cardiac fibroblasts

**FIGURE 7:**
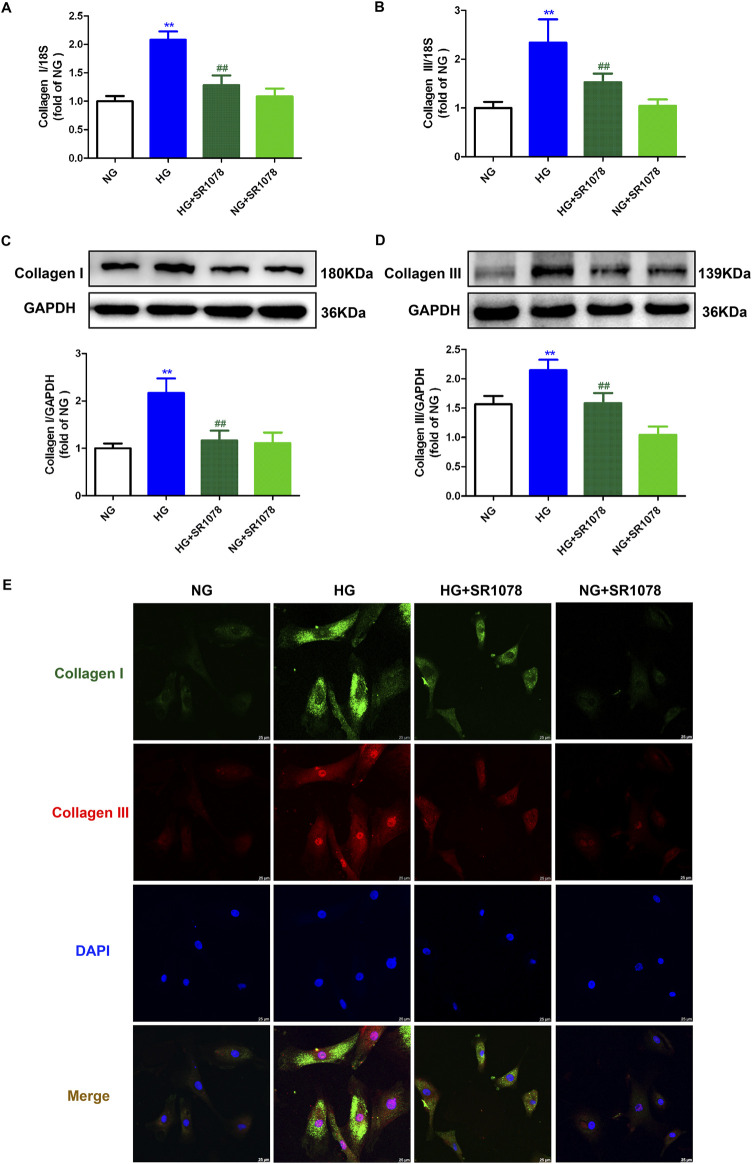
SR1078 reduced collagen synthesis in high glucose stimulated cardiac fibroblasts. After pre-administration with SR1078 (10 µM) for 4 h, the cardiac fibroblasts were stimulated with normal glucose (NG, glucose 5.5 mM and mannitol 27.8 mM) or high glucose (HG, 33.3 mM) for 48 h **(A, B)** The mRNA expressions of Collagen I and Collagen III were measured by Real-time PCR. **(C, D)** The protein expressions of Collagen I and Collagen III were measured by Western blot. GAPDH was serviced as a control. **(E)** Collagen I and collagen III were immunofluorescence stained with Alexa Fluor 488 (green) and Cy3 (red) conjugated IgG, respectively. The nuclei were stained with DAPI (blue). Bar = 25 μm ^**^ *p* < 0.01 verses NG; ^##^ *p* < 0.01 verses HG, n = 6.

Real-time-PCR, Western blot, and immunofluorescence demonstrated that enhanced Collagen I and III Collagen syntheses in high glucose stimulated cardiac fibroblasts were suppressed by SR1078 (Figure 7), suggesting that SR1078 reduced synthesis of collagen in these cells.

### 3.8 SR1078 suppresses oxidative stress in high glucose stimulated cardiac fibroblasts

**FIGURE 8:**
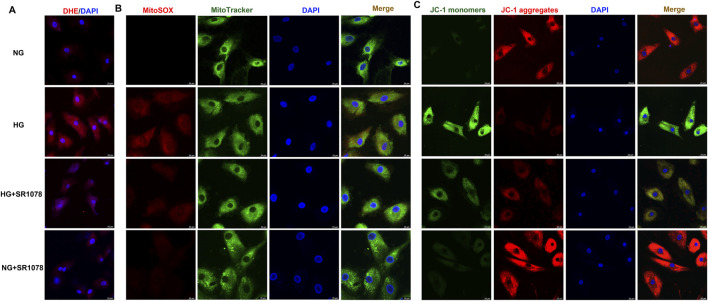
SR1078 suppressed oxidative stress in high glucose stimulated cardiac fibroblasts. After pre-administration with SR1078 (10 µM) for 4 h, the cardiac fibroblasts were stimulated with normal glucose (NG, glucose 5.5 mM and mannitol 27.8 mM) or high glucose (HG, 33.3 mM) for 48 h **(A)** DHE staining with red fluorescent was performed to measure superoxide anion production in cardiac fibroblasts. Bar = 25 μm. **(B)** MitoSOX staining with red fluorescent was performed to measure Mitochondrial ROS production. MitoTracker with green fluorescent was stained to co-localize Mitochondria. Bar = 25 μm. **(C)** Mitochondrial membrane potential was measured by JC-1 staining. Bar = 25 μm.

DHE staining showed that stronger red fluorescence was weakened by SR1078 in high glucose stimulated cardiac fibroblasts (Figure 8A), suggesting SR1078 inhibited cellular superoxide anion production in these cells. MitoSOX staining demonstrated that stronger MitoSOX fluorescence was attenuated by SR1078 in high glucose stimulated cardiac fibroblasts (Figure 8B), suggesting SR1078 suppressed mitochondrial ROS production in this context. JC-1 staining indicated that stronger green fluorescence was alleviated, while weaker red fluorescence was strengthened by SR1078 in high glucose stimulated cardiac fibroblasts (Figure 8C), suggesting that mitochondrial membrane potential was enhanced by SR1078. Taken together, SR1078 suppressed oxidative stress in high glucose stimulated cardiac fibroblasts.

### 3.9 SR1078 alleviates necroptosis in high glucose stimulated cardiac fibroblasts

**FIGURE 9:**
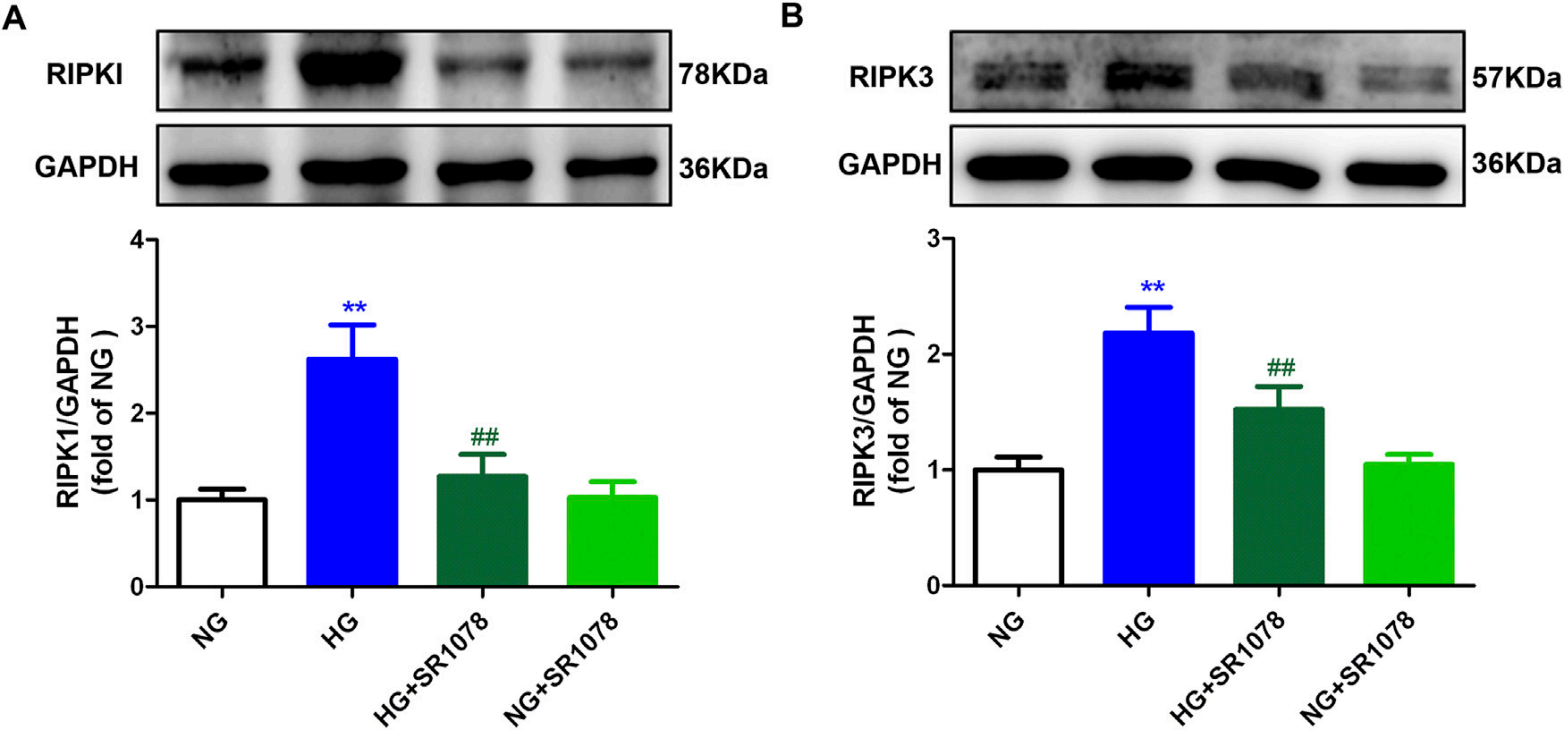
SR1078 alleviated necroptosis in high glucose stimulated cardiac fibroblasts. After pre-administration with SR1078 (10 µM) for 4 h, the cardiac fibroblasts were stimulated with normal glucose (NG, glucose 5.5 mM and mannitol 27.8 mM) or high glucose (HG, 33.3 mM) for 48 h. Expression of RIPK1 **(A)** and RIPK3 **(B)** protein was measured by Western blot. GAPDH was serviced as a control. ^**^ *p* < 0.01 verses NG; ^##^ *p* < 0.01 verses HG, n = 6.

Western blot showed that the increased RIPK3 and RIPK1 expressions were reduced by SR1078 in high glucose stimulated cardiac fibroblasts (Figure 9), suggesting SR1078 alleviated necroptosis.

## 4 Discussion

Our present study firstly investigated the effects and potential mechanisms underlying high glucose-stimulated cardiac fibroblast proliferation in the context of RORα antagonist and agonist. The data verified that activation of RORα, through the inhibition of necroptosis, exerts protective effects against cell proliferation, thereby proposing a novel approach to alleviate DC. Nonetheless, several limitations are present in the current study. Firstly, the level of RORα mRNA or protein in the myocardium of diabetic patients was not detected in the present study. Secondly, neonatal rat cardiac fibroblasts may not serve as an optimal model for assessing diabetes-induced alterations. Utilizing primary cardiac fibroblasts derived from diabetic adult mice would provide valuable insights into the roles of RORα in the pathological process of DC. Thirdly, in addition to high glucose, elevated fatty acids and insulin are also prevalent in the context of diabetes. High glucose alone may not sufficiently replicate the conditions associated with type 2 diabetes. Further studies should consider stimulation with high fatty acid and/or high insulin to more accurately reflect the diabetic environment.

Diabetes mellitus, a metabolic disorder, arises from insulin secretion deficiency or insulin dysfunction. In 2021, it was estimated that approximately 537 million individuals aged 20–79 were living with diabetes globally, with projections indicating an increase to 784 million by 2045 (Zhang S. et al., 2023; Huo et al., 2023). Chronic diabetes can cause various complications including nephropathy, retinal disorders, cardiovascular damage and peripheral neuropathy. Among these complications, DC is distinguished by its unique pathophysiological mechanisms, including early-stage abnormalities in diastolic function of the heart, ultimately progressing to clinical heart failure even in absence of coronary artery disease, hypertension and dyslipidemia (Seferović et al., 2024). The potential pathophysiological contributors to DC include immune dysfunction, impaired nutrient-sensing signaling, insulin resistance, cardiac inflammation, oxidative stress, subcellular component (primarily mitochondria) abnormalities, inappropriate activation of the renin-angiotensin system, and obesity (Huo et al., 2023; Zhang C. et al., 2024; Dhar et al., 2023; Hsuan et al., 2023). Collectively, these factors facilitate interstitial fibrosis of cardiac tissue, increase cardiac stiffness, and lead to subsequent systolic dysfunction, ultimately resulting in heart failure (Pan et al., 2023; Cheng et al., 2023). Despite the availability of various strategies to effectively manage blood glucose levels, the incidence of DC remains high, and progression to heart failure cannot be entirely prevented in certain patients (Parry et al., 2015; Kim et al., 2022). Consequently, seeking new means to delay or even halt the progression of DC is crucial for reducing the incidence and mortality associated with cardiovascular adverse events in individuals with diabetes.

Myocardial fibrosis, mainly resulting from an imbalance between ECM degradation and production, represents a significant manifestations of DC (Levick and Widiapradja, 2020). Myocardial fibrosis further exacerbates cardiac dysfunction and leads to distinct cardiovascular diseases. Activated myofibroblasts and fibroblasts serve as the principal sources of matrix proteins and act as the primary cellular effectors of myocardial fibrosis. Additionally, cardiomyocytes, vascular cells and immune cells can also attain fibrotic phenotypes in response to stress, ultimately causing the activation of fibroblast populations (Aguado-Alvaro et al., 2024). Various cytokines, including interleukin (IL)-1, IL-4, IL-6, IL-10, and tumor necrosis factor-α, along with neurohumoral pathways, and fibroblast growth factors such as platelet-derived growth factor and transforming growth factor-β can facilitate fibrotic signaling cascades by activating the downstream signaling pathways and interacting with surface receptors (Wang et al., 2023). Our present experiments confirmed that under high glucose (33.3 mM) stimulation, cardiac fibroblasts number was increased, collagen secretion was elevated, and cell proliferation was significantly accelerated, indicating a marked cardiac fibroblasts activation with pronounced fibrotic characteristics. Therefore, there is an urgent need for timely intervention to mitigate the progression of DC.

RORα, a member of orphan nuclear receptor family, exhibits higher tissue specificity and is involved in regulating processes of immunity, inflammation, circadian rhythms, and metabolic homeostasis. Notably, substantial evidence indicates that RORα influences both pathological and physiological within the cardiovascular system, including myocardial hypertrophy, hypertension, atherosclerosis, myocardial ischemia/reperfusion injury, and hypoxia or ischemia (Chen et al., 2023). Prior studies have demonstrated that RORα expression is downregulated in high glucose stimulated cardiomyocytes, as well as in the myocardium of diabetic mice. In streptozocin (STZ)-induced RORα knockout mice, exacerbated myocardial remodeling and cardiac dysfunction were observed, indicating a protective role for RORα against DC (Zhang S. et al., 2023; Zhao et al., 2017). However, the precise function of RORα in fibrosis remains to be elucidated. Furthermore, molecular mechanism underlying the transcriptional regulation pattern of RORα under a high glucose environment is still unclear. We previously found that hydrogen sulfide increased the expression of E2F transcription factor 1 (E2F1), promoted E2F1 binding to the promoter of RORα, increased RORα transcription, and eventually alleviated cell damage in cardiomyocytes with high glucose stimulation via a RORα-dependent manner (Zhang S. et al., 2023). Nevertheless, as a gasotransmitter, the potential of hydrogen sulfide as an effective regulatory molecule for RORα is still not optimistic. Fortunately, recent discoveries of endogenous ligands of RORα suggest that pharmacological modulation of RORα expression or activity through the use of exogenous agonists or antagonists may allow for the precise control of RORα within a physiological range, thereby maintaining the homeostasis of the cardiovascular system (Solt et al., 2011). RORα antagonist SR3335, which is actually one selective RORα inverse agonist of RORα, has demonstrated a substantial capacity to inhibit RORα activity upon its binding (Liang et al., 2021). In contrast, SR1078 functions as a RORα agonist, directly interacting with the ligand-binding domain of RORα, which increases the transcriptional activity of RORα target genes (Moreno-Smith et al., 2021). The present study found that inhibiting RORα activity further aggravated cell damage, increased cell number, upregulated collagen I and collagen III secretion, enhanced EdU-staining positive cells, and elevated PCNA and α-SMA expressions in high glucose stimulated cardiac fibroblasts. Conversely, activating RORα activity mitigates the above manifestations, indicating that adjusting RORα activity through pharmacological means represents an effective strategy for regulating cardiac fibroblasts proliferation with high glucose stimulation.

The pathogenic mechanism underlying DC remain incompletely elucidated, with associations identified between DC and cardiac metabolic disorders, microvascular dysfunction, endoplasmic reticulum stress, inflammation, mitochondrial dysfunction, oxidative stress, impaired Ca^2+^ handling, and apoptosis (Hsuan et al., 2023). Moreover, as of now, the precise protective mechanism of RORα on cardiovascular system is yet to be fully clarified (Chen et al., 2023). Necroptosis is a novel and unique form of regulated and programmed cell death (Newton et al., 2024). Necroptosis is primarily governed by receptor-binding protein kinases (notably RIPK1 and RIPK3). This process involves the sequential activation and phosphorylation of key proteins of necroptosis, culminating in the disruption of plasma membrane integrity and the amplification of inflammatory responses, which contribute to cellular dysfunction (Yuan and Ofengeim, 2024). Specifically, necroptosis may be triggered by various stimuli, predominantly tumor necrosis factor (TNF). In the absence of caspase-8, RIPK1 undergoes auto-phosphorylation at its serine/threonine residue sites and combines to RIPK3 through RIP homotypic interaction motif (RHIM), forming a RIPK1-RIPK3 complex named as necrosome. This complex subsequently recruits and activates the downstream protein MLKL, which is then phosphorylated. Then, phosphorylated MLKL translocates to the cell membrane, resulting in membrane rupture and the release of damage-associated molecular patterns (DAMPs), thereby mediating the occurrence of necroptosis (Aguado-Alvaro et al., 2024; Chaouhan et al., 2022). Importantly, as DC progresses, mitochondrial dysfunction is further exacerbated to enhance oxidative stress, which in turn promotes the process of necroptosis and the release of cellular contents to speed up cardiac fibroblasts’ proliferation and synthesis of collagen. Under high glucose stimulation, mitochondrial membrane permeability alters to enhance ROS production and the occurrence of necroptosis, thereby increasing the possibility of oxidative stress burs. In turn, ROS prone to leading to mitochondrial dysfunction and cardiac fibroblasts proliferation, accelerating the myocardial fibrosis during the process of DC (Song et al., 2021; Gong et al., 2022; Zhang S. et al., 2023). That is to say, the mechanism of excessive cardiac fibroblasts proliferation in DC might be attributed to oxidative stress and necroptosis. Our research verified that the inhibition of RORα activity resulted in enhanced oxidative stress levels, reduced mitochondrial membrane potential, promoted necroptosis, and subsequently accelerated cardiac fibroblasts proliferation with high glucose stimulation. Conversely, the enhancement of RORα activity reversed the above manifestations, suggesting that necroptosis and RORα-mediated inhibition of oxidative stress may constitute a protective mechanism regulating the proliferation of cardiac fibroblasts. Interestingly, our study showed that SR1078 attenuated necroptosis while simultaneously inhibiting cell proliferation, a seemingly paradoxical outcome. However, it is plausible that following necroptosis, cardiac fibroblasts release additional cellular contents due to membrane rupture, thereby promoting the proliferation of cardiac fibroblast (Zhang et al., 2021). Thereby the inhibitory effects of SR1078 on cardiac fibroblast proliferation may be ascribed to its capacity to alleviate necroptosis.

In summary, RORα antagonist SR3335 promoted cell injury and proliferation, enhanced collagen synthesis, facilitated necroptosis and oxidative stress in high glucose stimulated cardiac fibroblasts. In contrast, RORα agonist SR1078 attenuated cell injury and proliferation, reduced collagen synthesis, alleviated necroptosis, and suppressed oxidative stress in high glucose stimulated cardiac fibroblasts. Our present study proposed RORα as a novel therapeutic target for addressing high glucose-induced cardiac fibroblasts proliferation, which is beneficial to clarify some other ideal therapeutic implication for DC.

## Data Availability

The raw data supporting the conclusions of this article will be made available by the authors, without undue reservation.

## References

[B1] Aguado-Alvaro L. P. Garitano N. Pelacho B. (2024). Fibroblast diversity and epigenetic regulation in cardiac fibrosis. Int. J. Mol. Sci. 25 (11), 6004. 10.3390/ijms25116004 38892192 PMC11172550

[B2] Cai K. Jiang H. Zou Y. Song C. Cao K. Chen S. (2024). Programmed death of cardiomyocytes in cardiovascular disease and new therapeutic approaches. Pharmacol. Res. 206, 107281. 10.1016/j.phrs.2024.107281 38942341

[B3] Cao L. Mu W. (2021). Necrostatin-1 and necroptosis inhibition: pathophysiology and therapeutic implications. Pharmacol. Res. 163, 105297. 10.1016/j.phrs.2020.105297 33181319 PMC7962892

[B4] Chaouhan H. S. Vinod C. Mahapatra N. Yu S. H. Wang I. K. Chen K. B. (2022). Necroptosis: a pathogenic negotiator in human diseases. Int. J. Mol. Sci. 23 (21), 12714. 10.3390/ijms232112714 36361505 PMC9655262

[B5] Chen D. Mo F. Liu M. Liu L. Xing J. Xiao W. (2024a). Characteristics of splenic PD-1^+^ γδT cells in Plasmodium yoelii nigeriensis infection. Immunol. Res. 72 (3), 383–394. 10.1007/s12026-023-09441-w 38265549 PMC11217126

[B6] Chen S. Li Q. Shi H. Li F. Duan Y. Guo Q. (2024b). New insights into the role of mitochondrial dynamics in oxidative stress-induced diseases. Biomed. Pharmacother. 178, 117084. 10.1016/j.biopha.2024.117084 39088967

[B7] Chen Y. Zhang S. P. Gong W. W. Zheng Y. Y. Shen J. R. Liu X. (2023). Novel therapeutic potential of retinoid-related orphan receptor α in cardiovascular diseases. Int. J. Mol. Sci. 24 (4), 3462. 10.3390/ijms24043462 36834872 PMC9959049

[B8] Cheng Y. Wang Y. Yin R. Xu Y. Zhang L. Zhang Y. (2023). Central role of cardiac fibroblasts in myocardial fibrosis of diabetic cardiomyopathy. Front. Endocrinol. (Lausanne) 14, 1162754. 10.3389/fendo.2023.1162754 37065745 PMC10102655

[B9] Dhar A. Venkadakrishnan J. Roy U. Vedam S. Lalwani N. Ramos K. S. (2023). A comprehensive review of the novel therapeutic targets for the treatment of diabetic cardiomyopathy. Ther. Adv. Cardiovasc. Dis. 17, 17539447231210170. 10.1177/17539447231210170 38069578 PMC10710750

[B10] Drăgoi C. M. Diaconu C. C. Nicolae A. C. Dumitrescu I. B. (2024). Redox homeostasis and molecular biomarkers in precision therapy for cardiovascular diseases. Antioxidants (Basel) 13 (10), 1163. 10.3390/antiox13101163 39456418 PMC11504313

[B11] Frangogiannis N. G. (2022). Transforming growth factor-β in myocardial disease. Nat. Rev. Cardiol. 19 (7), 435–455. 10.1038/s41569-021-00646-w 34983937

[B12] Gong W. Zhang S. Chen Y. Shen J. Zheng Y. Liu X. (2022). Protective role of hydrogen sulfide against diabetic cardiomyopathy via alleviating necroptosis. Free Radic. Biol. Med. 181, 29–42. 10.1016/j.freeradbiomed.2022.01.028 35101564

[B13] González A. López B. Ravassa S. San José G. Latasa I. Butler J. (2024). Myocardial interstitial fibrosis in hypertensive heart disease: from mechanisms to clinical management. Hypertension 81 (2), 218–228. 10.1161/HYPERTENSIONAHA.123.21708 38084597

[B14] He Z. Zhu Y. Ma H. Shen Q. Chen X. Wang X. (2024). Hydrogen sulfide regulates macrophage polarization and necroptosis to accelerate diabetic skin wound healing. Int. Immunopharmacol. 132, 111990. 10.1016/j.intimp.2024.111990 38574702

[B15] Hsuan C. F. Teng S. I. F. Hsu C. N. Liao D. Chang A. J. Lee H. L. (2023). Emerging therapy for diabetic cardiomyopathy: from molecular mechanism to clinical practice. Biomedicines 11 (3), 662. 10.3390/biomedicines11030662 36979641 PMC10045486

[B16] Huo J. L. Feng Q. Pan S. Fu W. J. Liu Z. Liu Z. (2023). Diabetic cardiomyopathy: early diagnostic biomarkers, pathogenetic mechanisms, and therapeutic interventions. Cell Death Discov. 9 (1), 256. 10.1038/s41420-023-01553-4 37479697 PMC10362058

[B17] Khan I. Yousif A. Chesnokov M. Hong L. Chefetz I. (2021). A decade of cell death studies: breathing new life into necroptosis. Pharmacol. Ther. 220, 107717. 10.1016/j.pharmthera.2020.107717 33164841

[B18] Kim A. H. Jang J. E. Han J. (2022). Current status on the therapeutic strategies for heart failure and diabetic cardiomyopathy. Biomed. Pharmacother. 145, 112463. 10.1016/j.biopha.2021.112463 34839258

[B19] Koerner L. Wachsmuth L. Kumari S. Schwarzer R. Wagner T. Jiao H. (2024). ZBP1 causes inflammation by inducing RIPK3-mediated necroptosis and RIPK1 kinase activity-independent apoptosis. Cell Death Differ. 31 (7), 938–953. 10.1038/s41418-024-01321-6 38849574 PMC11239871

[B20] Levick S. P. Widiapradja A. (2020). The diabetic cardiac fibroblast: mechanisms underlying phenotype and function. Int. J. Mol. Sci. 21 (3), 970. 10.3390/ijms21030970 32024054 PMC7036958

[B21] Li Y. Liu X. Wan L. Han B. Ma S. Pan H. (2023). Metformin suppresses cardiac fibroblast proliferation under high-glucose conditions via regulating the mitochondrial complex I protein Grim-19 involved in the Sirt1/Stat3 signaling pathway. Free Radic. Biol. Med. 206, 1–12. 10.1016/j.freeradbiomed.2023.06.013 37353174

[B22] Liang T. Chen T. Qiu J. Gao W. Qiu X. Zhu Y. (2021). Inhibition of nuclear receptor RORα attenuates cartilage damage in osteoarthritis by modulating IL-6/STAT3 pathway. Cell Death Dis. 12 (10), 886. 10.1038/s41419-021-04170-0 34584074 PMC8478978

[B23] Liu S. Perez P. Sun X. Chen K. Fatirkhorani R. Mammadova J. (2024b). MLKL polymerization-induced lysosomal membrane permeabilization promotes necroptosis. Cell Death Differ. 31 (1), 40–52. 10.1038/s41418-023-01237-7 37996483 PMC10782024

[B24] Liu T. Hao Y. Zhang Z. Zhou H. Peng S. Zhang D. (2024a). Advanced cardiac patches for the treatment of myocardial infarction. Circulation 149 (25), 2002–2020. 10.1161/CIRCULATIONAHA.123.067097 38885303 PMC11191561

[B25] Lu Q. B. Fu X. Liu Y. Wang Z. C. Liu S. Y. Li Y. C. (2023). Disrupted cardiac fibroblast BCAA catabolism contributes to diabetic cardiomyopathy via a periostin/NAP1L2/SIRT3 axis. Cell. Mol. Biol. Lett. 28 (1), 93. 10.1186/s11658-023-00510-4 37993768 PMC10666354

[B26] Moreno-Smith M. Milazzo G. Tao L. Fekry B. Zhu B. Mohammad M. A. (2021). Restoration of the molecular clock is tumor suppressive in neuroblastoma. Nat. Commun. 12 (1), 4006. 10.1038/s41467-021-24196-4 34183658 PMC8238982

[B27] Newton K. Strasser A. Kayagaki N. Dixit V. M. (2024). Cell death. Cell 187 (2), 235–256. 10.1016/j.cell.2023.11.044 38242081

[B28] Pan K. L. Hsu Y. C. Chang S. T. Chung C. M. Lin C. L. (2023). The role of cardiac fibrosis in diabetic cardiomyopathy: from pathophysiology to clinical diagnostic tools. Int. J. Mol. Sci. 24 (10), 8604. 10.3390/ijms24108604 37239956 PMC10218088

[B29] Parry H. M. Deshmukh H. Levin D. Van Zuydam N. Elder D. H. Morris A. D. (2015). Both high and low HbA1c predict incident heart failure in type 2 diabetes mellitus. Circ. Heart Fail. 8 (2), 236–242. 10.1161/CIRCHEARTFAILURE.113.000920 25561089 PMC4366571

[B30] Sajinovic T. Baier G. (2023). New insights into the diverse functions of the NR2F nuclear orphan receptor family. Front. Biosci. (Landmark Ed). 28 (1), 13. 10.31083/j.fbl2801013 36722280

[B31] Seferović P. M. Paulus W. J. Rosano G. Polovina M. Petrie M. C. Jhund P. S. (2024). Diabetic myocardial disorder. A clinical consensus statement of the heart failure association of the ESC and the ESC working group on myocardial and pericardial diseases. Eur. J. Heart Fail 26 (9), 1893–1903. 10.1002/ejhf.3347 38896048

[B32] Shao Y. Wang X. Zhou Y. Jiang Y. Wu R. Lu C. (2021). Pterostilbene attenuates RIPK3-dependent hepatocyte necroptosis in alcoholic liver disease via SIRT2-mediated NFATc4 deacetylation. Toxicology 461, 152923. 10.1016/j.tox.2021.152923 34474091

[B33] Shen Y. Tang Q. Wang J. Zhou Z. Yin Y. Zhang Y. (2023). Targeting RORα in macrophages to boost diabetic bone regeneration. Cell Prolif. 56 (10), e13474. 10.1111/cpr.13474 37051760 PMC10542986

[B34] Sheng S. Y. Li J. M. Hu X. Y. Wang Y. (2023). Regulated cell death pathways in cardiomyopathy. Acta Pharmacol. Sin. 44 (8), 1521–1535. 10.1038/s41401-023-01068-9 36914852 PMC10374591

[B35] Solt L. A. Kumar N. Nuhant P. Wang Y. Lauer J. L. Liu J. (2011). Suppression of TH17 differentiation and autoimmunity by a synthetic ROR ligand. Nature 472 (7344), 491–494. 10.1038/nature10075 21499262 PMC3148894

[B36] Song S. Ding Y. Dai G. L. Zhang Y. Xu M. T. Shen J. R. (2021). Sirtuin 3 deficiency exacerbates diabetic cardiomyopathy via necroptosis enhancement and NLRP3 activation. Acta Pharmacol. Sin. 42 (2), 230–241. 10.1038/s41401-020-0490-7 32770173 PMC8027053

[B37] Tian J. Zhang M. Suo M. Liu D. Wang X. Liu M. (2021). Dapagliflozin alleviates cardiac fibrosis through suppressing EndMT and fibroblast activation via AMPKα/TGF-β/Smad signalling in type 2 diabetic rats. J. Cell. Mol. Med. 25 (16), 7642–7659. 10.1111/jcmm.16601 34169635 PMC8358881

[B38] Wahyuni T. Kobayashi A. Tanaka S. Miyake Y. Yamamoto A. Bahtiar A. (2021). Maresin-1 induces cardiomyocyte hypertrophy through IGF-1 paracrine pathway. Am. J. Physiol. Cell Physiol. 321 (1), C82–C93. 10.1152/ajpcell.00568.2020 34038245

[B39] Wang Q. Spurlock B. Liu J. Qian L. (2023). Fibroblast reprogramming in cardiac repair. JACC Basic Transl. Sci. 9 (1), 145–160. 10.1016/j.jacbts.2023.06.012 38362341 PMC10864899

[B40] Xiong J. Wang Z. Cao J. Dong Y. Chen Y. (2020). Melatonin mediates monochromatic light-induced proliferation of T/B lymphocytes in the spleen via the membrane receptor or nuclear receptor. Poult. Sci. 99 (9), 4294–4302. 10.1016/j.psj.2020.06.008 32867973 PMC7598018

[B41] Yu W. Wang L. Ren W. Y. Xu H. X. Wu N. N. Yu D. H. (2024). SGLT2 inhibitor empagliflozin alleviates cardiac remodeling and contractile anomalies in a FUNDC1-dependent manner in experimental Parkinson's disease. Acta Pharmacol. Sin. 45 (1), 87–97. 10.1038/s41401-023-01144-0 37679644 PMC10770167

[B42] Yuan J. Ofengeim D. (2024). A guide to cell death pathways. Nat. Rev. Mol. Cell Biol. 25 (5), 379–395. 10.1038/s41580-023-00689-6 38110635

[B43] Zhang C. Shi Y. Liu C. Sudesh S. M. Hu Z. Li P. (2024b). Therapeutic strategies targeting mechanisms of macrophages in diabetic heart disease. Cardiovasc Diabetol. 23 (1), 169. 10.1186/s12933-024-02273-4 38750502 PMC11097480

[B44] Zhang J. Cao J. Qian J. Gu X. Zhang W. Chen X. (2023a). Regulatory mechanism of CaMKII δ mediated by RIPK3 on myocardial fibrosis and reversal effects of RIPK3 inhibitor GSK'872. Biomed. Pharmacother. 166, 115380. 10.1016/j.biopha.2023.115380 37639745

[B45] Zhang J. Qian J. Zhang W. Chen X. (2023b). The pathophysiological role of receptor-interacting protein kinase 3 in cardiovascular disease. Biomed. Pharmacother. 165, 114696. 10.1016/j.biopha.2023.114696 37329707

[B46] Zhang Q. Wang L. Wang S. Cheng H. Xu L. Pei G. (2022b). Signaling pathways and targeted therapy for myocardial infarction. Signal Transduct. Target Ther. 7 (1), 78. 10.1038/s41392-022-00925-z 35273164 PMC8913803

[B47] Zhang S. Shen J. Zhu Y. Zheng Y. San W. Cao D. (2023c). Hydrogen sulfide promoted retinoic acid-related orphan receptor α transcription to alleviate diabetic cardiomyopathy. Biochem. Pharmacol. 215, 115748. 10.1016/j.bcp.2023.115748 37591449

[B48] Zhang Y. Gong W. Xu M. Zhang S. Shen J. Zhu M. (2021). Necroptosis inhibition by hydrogen sulfide alleviated hypoxia-induced cardiac fibroblasts proliferation via sirtuin 3. Int. J. Mol. Sci. 22 (21), 11893. 10.3390/ijms222111893 34769322 PMC8584899

[B49] Zhang Y. Lu P. Jin S. Zhang J. Chen X. (2024a). Transcriptional activation of SIRT5 by FOXA1 reprograms glycolysis to facilitate the malignant progression of diffuse large B-cell lymphoma. Cell. Signal. 123, 111356. 10.1016/j.cellsig.2024.111356 39173857

[B50] Zhang Y. Wang Z. Dong Y. Cao J. Chen Y. (2022a). Melatonin nuclear receptors mediate green-and-blue-monochromatic-light-combinations-inhibited B lymphocyte apoptosis in the bursa of chickens via reducing oxidative stress and Nfκb expression. Antioxidants (Basel) 11 (4), 748. 10.3390/antiox11040748 35453433 PMC9029876

[B51] Zhao Y. Xu L. Ding S. Lin N. Ji Q. Gao L. (2017). Novel protective role of the circadian nuclear receptor retinoic acid-related orphan receptor-α in diabetic cardiomyopathy. J. Pineal Res. 62 (3), 12378. 10.1111/jpi.12378 27862268

[B52] Zheng Y. Xu Y. Ji L. San W. Shen D. Zhou Q. (2024). Roles of distinct nuclear receptors in diabetic cardiomyopathy. Front. Pharmacol. 15, 1423124. 10.3389/fphar.2024.1423124 39114353 PMC11303215

[B53] Zhou Y. Jin H. Wu Y. Chen L. Bao X. Lu C. (2019). Gallic acid protects against ethanol-induced hepatocyte necroptosis via an NRF2-dependent mechanism. Toxicol. In Vitro. 57, 226–232. 10.1016/j.tiv.2019.03.008 30853489

[B54] Zhou Y. Xiang Y. Liu S. Li C. Dong J. Kong X. (2024). RIPK3 signaling and its role in regulated cell death and diseases. Cell Death Discov. 10 (1), 200. 10.1038/s41420-024-01957-w 38684668 PMC11059363

